# Streptococcal Toxic Shock Syndrome Due to Invasive Coloproctitis Caused by Group G Streptococcus: A Case Report and Literature Review

**DOI:** 10.7759/cureus.48589

**Published:** 2023-11-09

**Authors:** Nobuko Matsuoka, Takuya Kimura, Yoshitake Endo, Masanari Hamaguchi, Yoshitaka Ogata, Kiyoaki Uryu, Yoshinori Murao, Teruyoshi Amagai

**Affiliations:** 1 Department of Surgery/Department of Emergency Medicine, Yao Tokushukai General Hospital, Osaka, JPN; 2 Department of Surgery, Yao Tokushukai General Hospital, Osaka, JPN; 3 Department of Critical Care Medicine, Yao Tokushukai General Hospital, Osaka, JPN; 4 Emergency Department, Yao Tokushukai General Hospital, Osaka, JPN; 5 Faculty of Health Care Sciences, Department of Clinical Engineering, Jikei University of Health Care Sciences, Osaka, JPN

**Keywords:** invasive infection, intestinal infection, streptococcus dysgalactiae subsp. equisimillis (sdse), group g streptococcus (ggs), streptococcal toxic shock syndrome (stss)

## Abstract

*Streptococcus dysgalactiae subsp. equisimillis *(SDSE) is classified as a group G streptococcus (GGS). In systemic SDSE infection, septic shock is easily induced and has a high mortality of 44%. The case was a 78-year-old man presented with fever and chills of 20 hours duration. He was in shock at the presentation and developed melena on day nine. CT images showed bowel wall thickening with emphysema and bedside colonoscopy showed active bleeding in the descending colon and rectum. Blood cultures were positive for *Streptococcus dysgalactiae* and a diagnosis of streptococcal toxic shock syndrome (STSS) due to SDSE was made. Urgent Hartmann procedure with laparotomy for removal of descending and rectal colon was performed to relieve his shock status. His shock status was reversed after surgery. Surgical specimens confirmed the presence of SDSE on the intestinal mucosa. This is the first case of STSS due to SDSE infection of the intestinal wall. Resection of infected tissue in the setting of multiple organ dysfunction syndrome and necrotizing enterocolitis is indicated in such cases.

## Introduction

*Streptococci* are divided into 20 classifications (groups A to H and groups K to V) by Lancefield, based on the classification of sugars in the cell wall. *Streptococcus dysgalactiae* subsp. equisimillis (SDSE) is a *Streptococcus* strain classified in a group G streptococcus (GGS) [1]. SDSE has been identified as part of the normal flora of human skin, oral cavity, nasopharynx, gastrointestinal tract, and vagina. The concept of streptococcal toxic shock syndrome (STSS) was coined from "streptococcal toxic shock-like syndrome", first reported by Steven in 1989 in patients with scarlet fever associated with group A streptococcal infection [2]. STSS is easily induced in patients with systemic invasive SDSE infection and has a high mortality rate of 44%. We present the case of a 78-year-old male who developed STSS due to intestinal invasive SDSE infection. A review of PubMed and Google Scholar searches revealed no other cases of STSS infection originating in the gastrointestinal tract. We present a severe case of STSS that necessitates a surgical intervention.

## Case presentation

A 78-year-old man with a history of hypertension and cerebral infarction 10 years ago presented to the emergency department of our hospital with chief complaints of fever and chills of 10 hours duration. As he appeared to have no symptoms other than fever, he was sent home with acetaminophen as an antipyretic. He returned to the internal medicine outpatient clinic 20 hours after his initial presentation with complaints of dysuria and fever.

**Figure 1:**
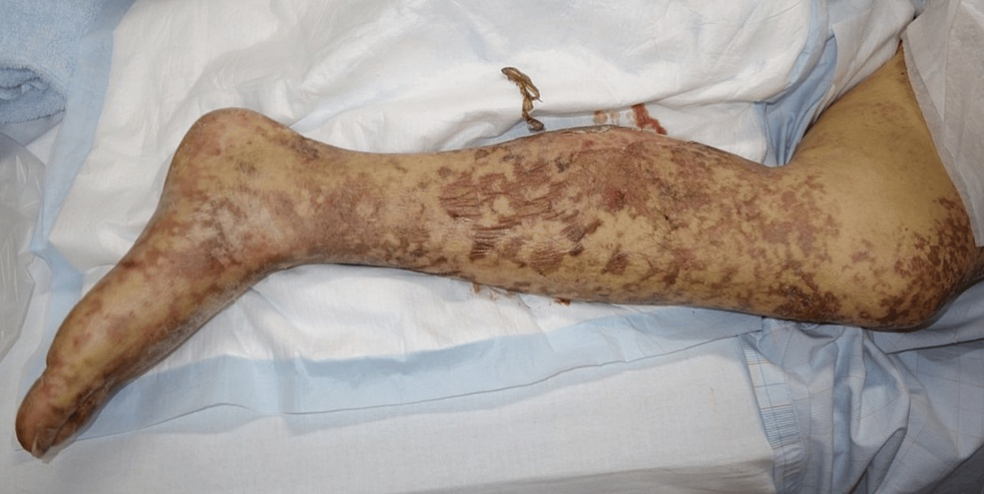
The erythematous rashes in the leg A reticular patch and erythematous rash appeared on the lower limb.

At the second presentation, the physical examination was as follows: weight 51.8 kg, body mass index (BMI) 17.9 kg/m^2^, GCS 15 by E4V5M6, body temperature 39.7°C, heart rate 128 beats/min, blood pressure 112/68 mmHg, respiratory rate 35 breaths/min, and SpO_2_ 92% on room air. A reticular rash not present on the first presentation now appeared on the trunk and all four limbs (Figure 1). No other abnormal findings in the oral cavity, chest, or abdomen were identified.

His blood findings were as follows: white blood cell (WBC) 5,300 counts/μL, C-reactive protein 24.22 mg/dL, procalcitonin > 100 ng/mL, blood urea nitrogen (BUN) 40.1 mg/dL, serum creatinine 3.08 mg/dL, aspartate aminotransferase (AST) 258 U/L, alanine aminotransferase (ALT) 118 U/L, lactate dehydrogenase (LDH) 698 U/L, lactic acid 41 mg/dl, prothrombin time-international normalized ratio (PT-INR) 2.18, D-dimer 39.9 μg/mL, and base excess -11 mEq/L. His urinalysis showed the presence of WBC, 5-9 counts/high power field, and bacteria 2.5×10^4 ^colonies/mL. His electrocardiography showed sinus rhythm and ultrasound cardiography showed normal heart function with a left ventricular ejection fraction of 68%. In addition, his plain abdominal computed tomography (CT) images showed no abnormal findings. In his culture studies, Gram-positive cocci in chains were isolated from two venous blood cultures. The antibiogram susceptibility studies for the blood isolated are seen in Table 1. The urine culture was negative for bacteria.

**Table 1:** Traditional antibiogram evaluating susceptibility to Streptococcus dysgalactiae subsp equisimilis Blood culture revealed the presence of *Streptococcus dysgalactiae* *subsp. equisimilis*. Antibiotic susceptibility testing for this pathogen was then performed. Abbreviations, ABPC: Ampicillin Sodium, AZM: Azithromycin, CAM: Clarithromycin, CCL: Cefaclor, CEZ: Cefazolin Sodium, CFPM: Cefepime, CLDM: Clindamycin Phosphate, CTM: Cefotiam, CTRX: Ceftriaxone Sodium, LVFX: Levofloxacin hydrate, MEPM: Meropenem Hydrate, MIC: Minimum Inhibitory Concentration, MINO: Minocycline Hydrochloride, IPM: Impenem / Cilastatin, PCG: Benzylpenicillin, S/ABPC: Sulbactam / Ampicillin, ST: Sulfa + Trimethoprim, VCM: Vancomycin.

Blood culture	Streptococcus dysgalactiae ssp equisimilis

Antibiotics	AZM	PCG	ABPC	CEZ	CTM	CFPM	CTRX	CCL	IPM
MIC	0.25	<=0.06	<=0.12	<=0.5	0.12	<=0.06	<=0.06	<=0.5	<=0.06
Sensitivity	S	S	S	S	S	S	S	S	S
Antibiotics	MEPM	S/ABPC	VCM	CAM	CLDM	MINO	LVFX	ST	
MIC	<=0.06	<=0.25	<=0.25	<=0.12	<=0.12	<=0.12	<=1	<=10	
Sensitivity	S	S	S	S	S	S	S	S	

**Figure 2:**
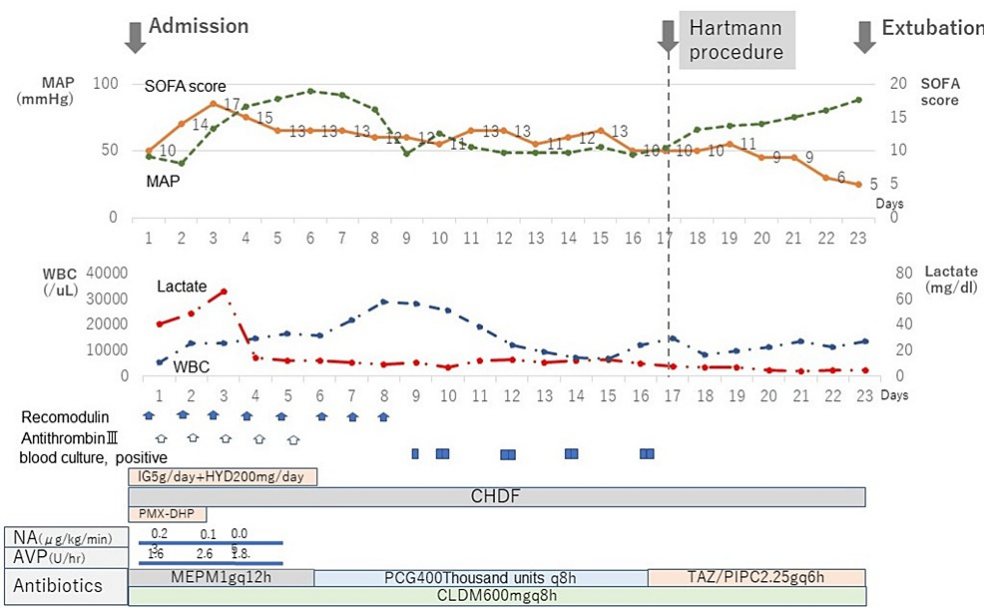
The timeline of the clinical course and treatments Top figure - the orange and green lines show the SOFA score and MAP, respectively. Bottom figure - the red and blue lines show serum lactate concentration and WBC, respectively. Abbreviations, AVP: arginine vasopressin, CHDF: continuous hemodiafiltration, CLDM: clindamycin, IG: immunoglobulin, HYD: hydrocortisone, MAP: mean arterial blood pressure, MEPM: meropenem, NA: noradrenalin, PCG: penicillin G, PMX-DHP: direct hemoperfusion with polymyxin B immobilized fiber, SOFA: sequential organ failure assessment, TAZ/PIPC: Tazobactam / Piperacillin, WBC: white blood cell.

During a physical examination in the outpatient clinic, he developed septic shock and was admitted to the intensive care unit after intubation and resuscitation. Within the first three hours, he developed multiple organ dysfunction syndrome (MODS) due to hepato-renal dysfunction and disseminated intravascular coagulation (DIC). Then, treatments started for MODS included intravenous administrations of antibacterial drugs (meropenem and clindamycin) and intravenous immunoglobulin (5 grams per day), continuous hemodiafiltration as endotoxin adsorption therapy (Figure 2). Endotoxin adsorption has the potential to reduce the biological cascade of Gram-negative sepsis and is available for the purpose of sepsis salvage.

**Figure 3:**
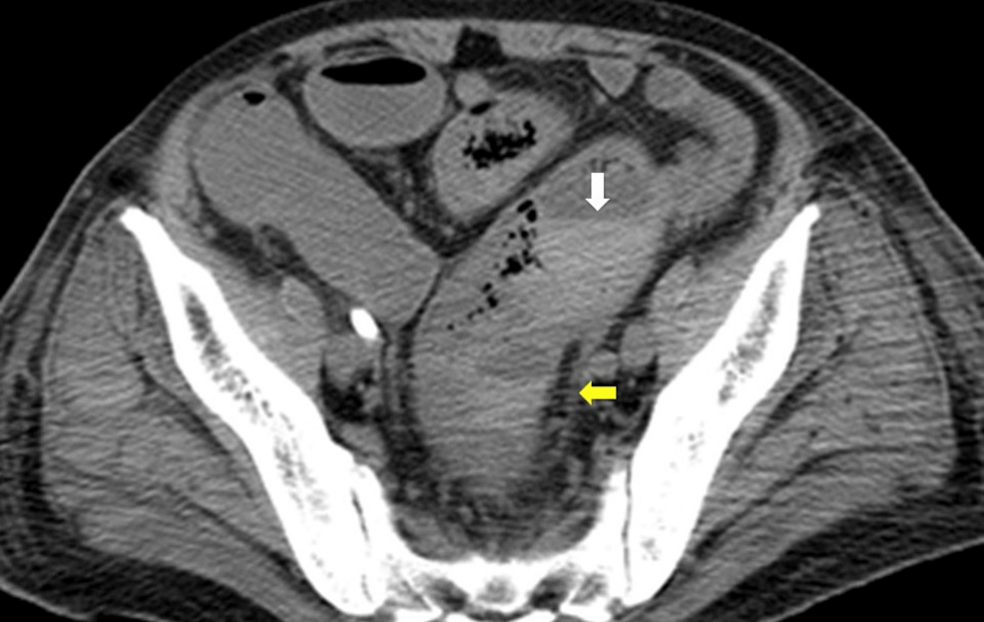
Abdominal computed tomography images Intramural emphysema was observed in the sigmoid colon (yellow arrow) and very bright intestinal fluid was observed (white arrow).

On day 6, Gram-positive cocci detected as a result of Progress bacterial cultures were proven to be *Streptococcus dysgalactiae,* and a diagnosis of STSS was made, followed by melena and shock before operation. A plain abdominal CT taken on day 15 showed marked thickening of the intestinal wall from the sigmoid colon to the rectum with intramural emphysema (Figure 3).

**Figure 4:**
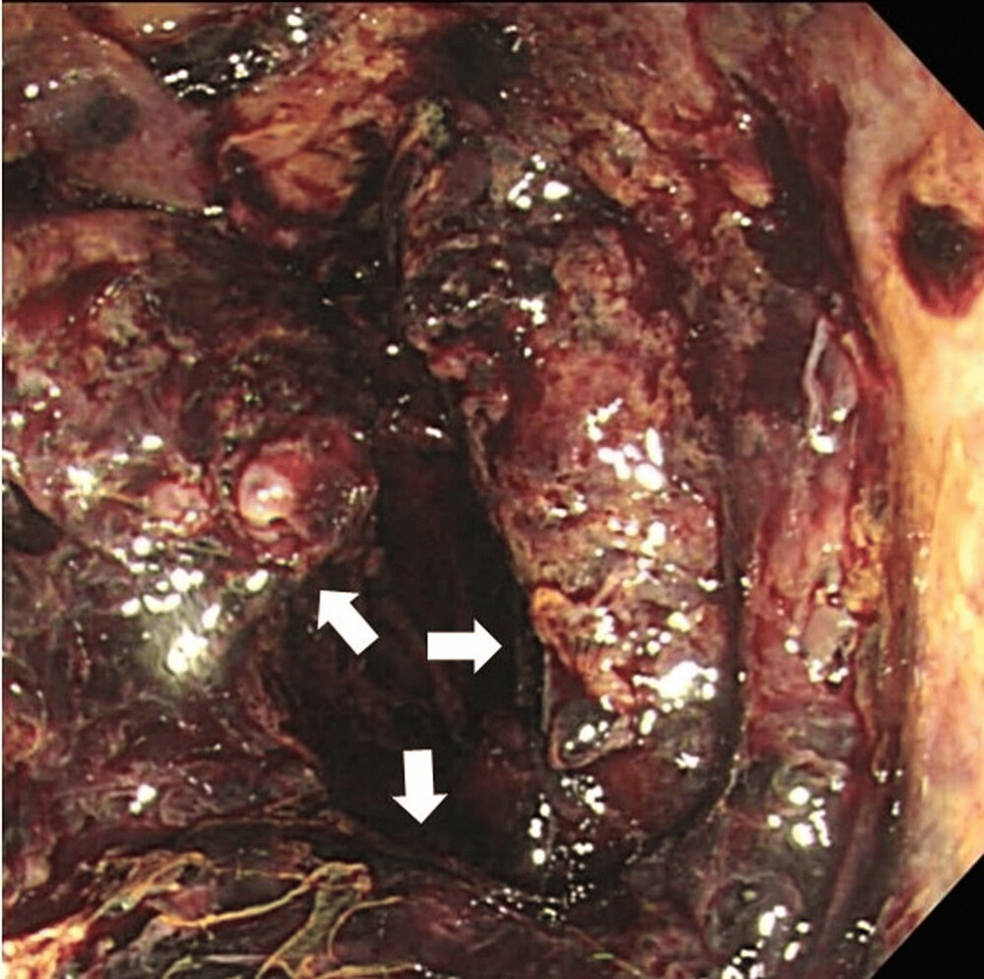
Colonoscopy photographs Multiple rectal ulcers with bleeding and sloughing of the mucosa are observed (white arrows).

In addition, a bedside colonoscopy on day 15 revealed sloughing of the rectal mucosa and multiple bleeding from multiple rectal ulcers (Figure 4).

**Figure 5:**
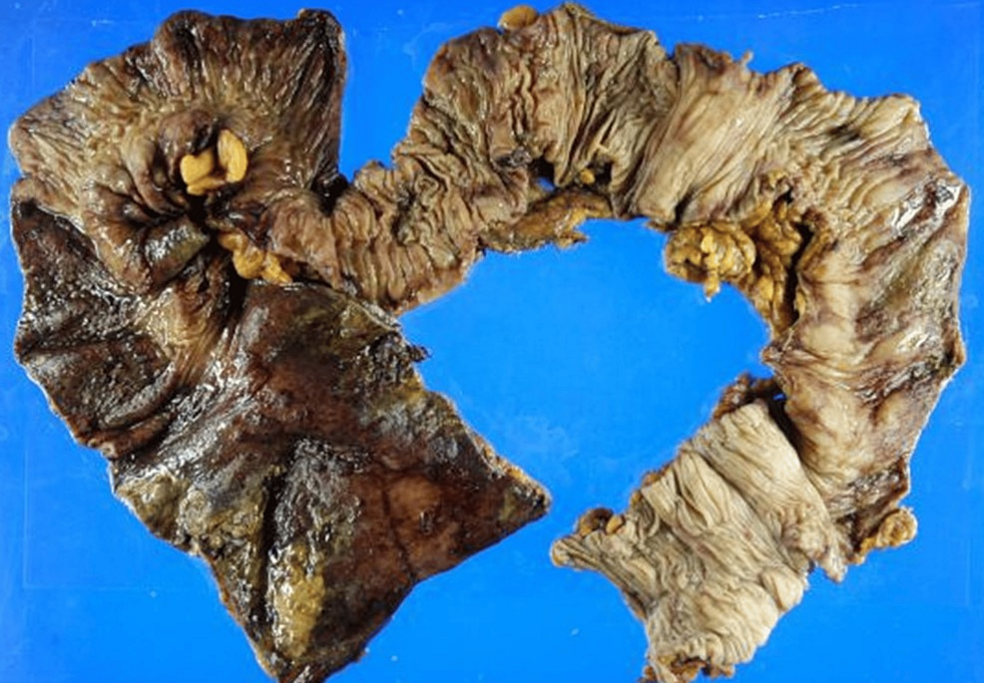
The surgical specimen from the descending colon to the rectum (naked-eye photography) The full-thickness necrosis of the rectum was seen. The left side and right of the photo are the rectum and descending colon, respectively.

**Figure 6:**
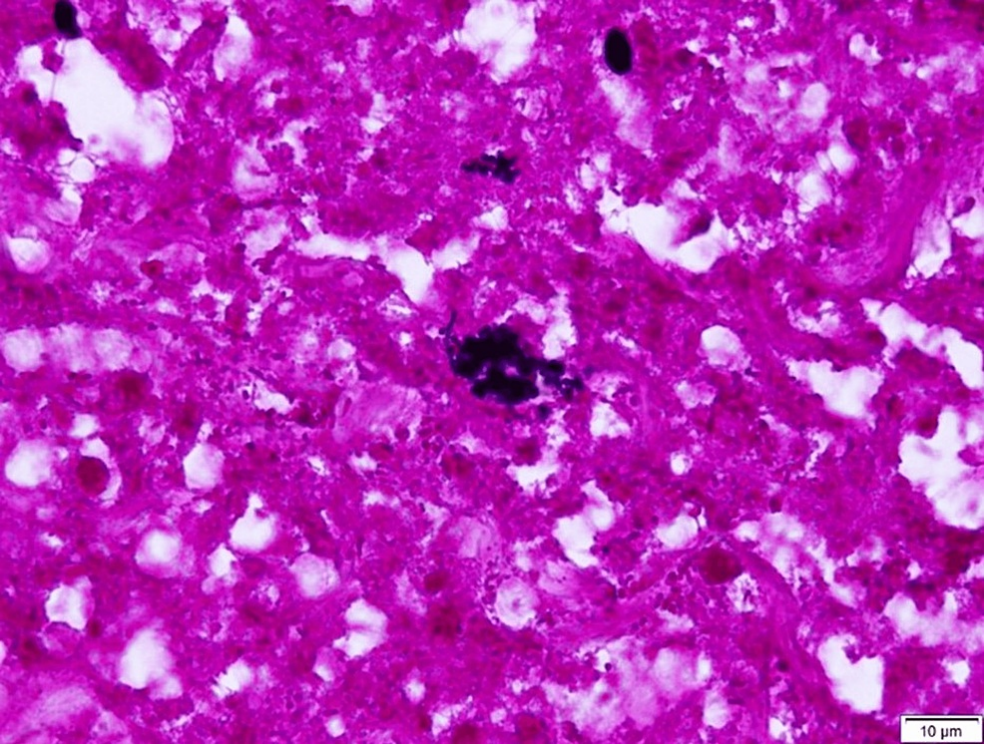
The microscopic image, Gram-positive staining (Micrograph, X400) There was an infiltration of inflammatory cells such as neutrophils, the wall structure was unclear, the mucosal structure was collapsed and there were some SDSE bacterial clumps in the intestinal mucosa.

Based on these findings, bowel necrosis was strongly suspected and bowel resection was considered necessary to reverse the hemorrhagic and septic shock and was performed. At this point, his general status seemed too poor to treat by medical options and we explained the risk of death during surgery to the family, obtained their consent, and decided to perform the surgery. On day 17, an urgent Hartmann procedure was performed to relieve his MODS status. The SOFA score, which was 17 to 13 points before surgery, gradually decreased and improved during the six days after surgery, recovering to five points, suggesting that the STSS treatment was effective. Pathologic findings of surgical specimens were severe hemorrhage in all layers taken from the sigmoid colon to the rectum (Figure 5) and the intestinal mucosa was accompanied by bacterial masses of *Streptococcus dysgalactiae* *subsp. equisimillis* (SDSE) (Figure 6). He was discharged from the hospital on day 102.

## Discussion

STSS caused by severe invasive *Streptococcus* species-induced septicemia

The SDSE has emerged as a critical cause of severe *Streptococcus* species-induced infections [3]. Among *Streptococcus* species-induced infection, severe invasive infection induces STSS. Patients with STSS are rarely opportunistic hosts and have a sudden onset of pyrexia. 

STSS definition and its clinical course of three phases

The definition of STSS by the Center for Disease Control and Prevention (CDC) is “an infection with *Streptococcus* pyogenes accompanied by sudden onset of shock, organ failure, and frequent death” [4]. Clinical manifestations of STSS include hypotension, multiorgan failure of the kidney and liver, coagulopathy, acute respiratory distress syndrome, and as a feature, a generalized erythematous macular rash and soft tissue necrosis may be the hallmark of SDSE-induced STSS. These clinical presentations are the results of inflammatory responses due to T cell activation by conjugating of exotoxin activating from superantigens with antigen processing cells (APC) [3].

Three phases of the clinical course of STSS

Prior to the onset of shock, STSS has three phases as follows: 1) the first phase shows influenza-like illness and skin manifestations, 2) the second phase has systemic manifestations including tachycardia, tachypnea, and high fever, 3) the third phase is circulatory shock [5]. The diagnosis of STSS in the early stages is too difficult to start medical treatment [6]. This is one of the reasons why the mortality rate of STSS is as high as 44%. When considering the treatment of patients with STSS, clindamycin resistance in GAS has been rapidly increasing and linezolid is a promising alternative adjunctive agent [7]. Intravenous immunoglobulin appears to be controversial [6]. However, the choice of antibiotics must take into account that the likelihood of invasive infection by antibiotic-resistant bacteria has increased from 8.6% to 21.6% in the most recent report [8].

The risk factors of STSS

The risk factors of STSS could lead to the correct diagnosis of STSS at earlier stages. The risk factors include 65 years of age or older, skin injury or breakdown, chronic illness such as alcohol use disorder, diabetes mellitus [9,10], cardiovascular diseases, malignancies, cirrhosis, immunosuppressive conditions, or skin diseases, cardiovascular diseases, malignancies, cirrhosis, immunosuppressive conditions, skin diseases [9], cirrhosis, bone and joint disease, and illicit drug abuse [1]. Of these reported risk factors, only older age is proved by evidence-based analysis [6].

Similarities and differences between STSS and toxic shock-like syndrome (TSLS)

When treating patients with STSS, toxic shock-like syndrome (TSLS) also must be considered. TSLS is caused by invasive Staphylococci infection with septic shock. TSLS is treated by empiric antibiotic treatment with such as meropenem and simultaneous respiratory and circulatory support must be quickly initiated. The similarities are that both STSS and TSLS are life-threatening and are included together in the differential diagnosis [11]. The differences include the efficacy of clindamycin (CLDM) in STSS but not in TSLS, and the presence of risk factors in TSLS but not in STSS other than older age. When treating patients with septic shock, we must consider these similarities and differences when considering STSS and TSLS as differential diagnoses. In our case, treated under multiple organ failure and septic shock, SDSE was identified in masses present in the wall of the gastrointestinal tract extending from the descending colon to the rectum. Surgical removal of the bacterial mass must be chosen to relieve the life-threatening shock. As far as we have been able to search the literature, there are no cases of this bacterium infecting the gastrointestinal tract. This case is believed to be the first and worth reporting.

## Conclusions

A 78-year-old man presented with fever and chills of 10 hours duration. He was in shock and developed melena on day 9. CT images and bedside colonoscopy showed bowel wall thickening and active bleeding in the descending colon and rectum, respectively. Blood cultures were positive for *Streptococcus dysgalactiae* and a diagnosis of STSS due to SDSE was made. A Hartmann procedure with laparotomy for descending and rectal colectomy was performed to relieve his shock status. Surgical specimens confirmed the presence of SDSE on the intestinal mucosa. This is the first case of STSS with intestinal wall SDSE. In our experience, surgical removal of infected tissue appears to be a treatment option.
